# Graphic Model of Virtual Teaching Supervision through Fuzzy Logic in Non-University Educational Centers

**DOI:** 10.3390/ijerph192416533

**Published:** 2022-12-09

**Authors:** Nuria Falla-Falcón, Eloy López-Meneses, Miguel-Baldomero Ramírez-Fernández, Esteban Vázquez-Cano

**Affiliations:** 1 Department of Philology and Translation, Area of French Philology, Pablo de Olavide University, 41013 Sevilla, Spain; 2 Department of Education and Social Psychology, Area of School Organization and Didactics, Pablo de Olavide University, 41013 Seville, Spain; 3 Ecotec University, Km 13.5 Vía Samborondón, Samborondón 092302, Guayas, Ecuador; 4 Didactics and School Organization, Faculty of Education, National Distance Education University (UNED), 28040 Madrid, Spain

**Keywords:** training, teaching innovation, quality of education, training supervision, fuzzy logic

## Abstract

This research analyzes the supervision of non-university virtual training due to the unexpected non-face-to-face teaching scenario caused by COVID-19 with a graphic model using the SULODITOOL^®^ instrument. It arises as a research line of the Chair of Education and Emerging Technologies, Gamification and Artificial Intelligence of the Pablo de Olavide University (Seville) and is developed under the auspices of other assessment instruments within the framework of the functions and attributions of the Education Inspectorate of Spain. The aforementioned instrument is made up of 10 weighted supervisory indicators using fuzzy logic. The aggregation of linguistic variables of 242 expert judges was performed using the probabilistic OR function and defuzzified using the area centroid method to calculate the aforementioned weights. Based on the innovative analytical and graphic methodology used to analyze the supervision of virtual teaching, both synchronous and asynchronous, it stands out from the results obtained that there are certain supervision indicators, such as the training design and the methodology used, which should be considered as factors key in all the scenarios studied (primary education, compulsory secondary education and post-compulsory education).

## 1. Introduction

The COVID-19 pandemic created significant disruptions to education provision, including restrictions on in-person learning and the proliferation of remote learning. Little is known about how these interruptions were experienced by older adolescents and young adults [1]. In this scenario, the demand for quality in education is necessary; as a concept full of difficulties that must be defined, what is considered good learning must be characterized [2]. For this reason, it must be taken into account that when using an e-learning evaluation instrument not explicitly tailored to Massive Open Online Course (onwards MOOCs) [3], they should share common features with online courses.

The new way of learning through MOOCs is undoubtedly a milestone in education of the 21st century and has brought about a revolution in the continuing education model [4]. Information and communication technologies have revolutionized the world as it was known before its use and applicability to daily life. Their use has also moved to the educational field and has transformed the way people learn and teach today. In the midst of this panorama, massive open online courses emerge as an opportunity available for everyone to learn, which has caused many changes in the educational field. Among the main advantages are its free nature, the establishment of collaboration networks and time flexibility, while among the disadvantages we can highlight dropout, that some courses are not adapted or available for simple devices, or lack of follow-up. The main idea is to encourage the advantages of virtual training, given the great benefits they bring to education and in turn try to resolve the disadvantages that have been seen to lead to the use of MOOCs, in order to promote the effectiveness of their use [5].

MOOCs are based on connectivism, an epistemological system that provides ideas about how certain learning phenomena occur among connected students, but lacks the nature and structure of a theory [6]. That is why, among the disadvantages is the lack of harmonization of studies that provides a holistic view of the aggregation of indicators to improve student participation in MOOCs. Indeed, the coronavirus pandemic has accelerated the adoption of MOOCs, and student engagement has become even more essential to the success of this educational innovation. Therefore, it is necessary to examine the existing literature to derive important indicators to improve student participation in MOOC learning environments [7].

The quality of virtual training is an emerging field for researchers. The studies analyzed focus on the assessment of the pedagogical value of training through the Internet and its improvement in this field [8]. Thus, it does not seem so evident that MOOCs offer quality training [9] and improvement is necessary if they are to become a disruptive milestone [10].

Along these lines, the so-called t-MOOCs tend to rely on the completion of tasks by the student. The presence of technologies in educational tasks means that the skills that teachers must possess are broader than the mere mastery of content and teaching methodologies, which is why it is necessary to emphasize the development of digital teaching skills [11].

In this sense, of the standards and consortia developed for the quality of virtual courses [12], the use of the AENOR standard (Spanish Association for Standardization and Certification) has been chosen for this research. Its main contribution to the field of e-learning instruments is the [13] standard for quality management of virtual training. AENOR is currently a member of the ISO (International Organization for Standardization). This standard was updated in 2012 and contains several quality indicator headings.

Therefore, an investigation is necessary to detect the possible points of supervision of virtual teaching for non-university teaching, with the aim of improving and guaranteeing learning that is adjusted to quality non-face-to-face teaching and that ensures very little difference with the face-to-face teaching modality for users.

## 2. Literature Review

For the elaboration of a proposal of indicators to assess the pedagogical quality of open and massive online courses reflected in a three-dimensional model, as in the case of uMuMooc, a proposal of indicators for considering and assessing the pedagogical quality of MOOCs in the context of Murcia University was brought forward [14]. The intention was to contribute to the construction of parameters that imply an improvement in quality, the promotion of educational innovation processes using ICT, the development and dissemination of good teaching practices, and teacher training.

From this perspective, the theoretical context of the study requires describing the most relevant aspects of the ADECUR^®^ instrument, the [13] standard on quality management of virtual training, the EduTool^®^ instrument and the key aspects of the functions and attributions of the Education Inspectorate in Spain, in symbiosis with the premises of fuzzy logic in educational settings. Each one of them is detailed below:

### 2.1. The ADECUR^®^ Assessment Instrument

ADECUR^®^ is an evaluation instrument capable of analyzing and identifying the defining features of the didactic quality of virtual courses, from the scales provided by the socio-constructivist and investigative paradigm, as a way to promote the adequate development of teaching innovation processes [11]. Said instrument, with a registered trademark at the Spanish Patent and Trademark Office (current file number: 2855153), is the result of a doctoral thesis by Dr. D. Eloy López Meneses entitled “*Analysis of teaching models and strategies of teaching in Tele-training: design and experimentation of an evaluation instrument of the teaching strategies of telematic courses of university training*”, awarded the extraordinary prize of doctorate 2008/09, granted by the University of Seville. In this sense, the tool tries to analyze teaching models presented by virtual training courses and teaching strategies that are revealed by the presence of certain structural and organizational criteria. With this, the most pragmatic field of didactics prevails within a framework of theoretical reflection. This instrument investigates discovering and investigating the approach to the didactic model that underlies any virtual training course linked to the university environment and, in another sense, it also analyzes the teaching strategies of online university. This didactic instrument consists of two large dimensions: the psycho-didactic and technical aspects.

### 2.2. The UNE 66181:2012 Standard

In the last decade, the phenomenon of virtual training has been developed extraordinarily, propitiated by globalization and the development of information and communication technologies (hereinafter ICT), which has contributed to the improvement and expansion of the existing educational offering. The extraordinary increase in this virtual training offer is mainly due to two key success factors. On the one hand, there is a growing existence of teaching materials made available to users and, on the other, there is ease of access to educational markets, both for providers and for applicants. The standard [13] identifies the characteristics of the training actions in remote teaching and, in this way, users select the training that meets their needs. Thus, educational organizations can improve their offers of virtual training to reduce the possible difference between the needs of students and their levels of satisfaction. Therefore, through gains in reliability and credibility, virtual training can mitigate the risk of user abandonment and provide remote courses guaranteed by quality parameters.

There are ten relevant aspects that should be present in a MOOC model that are defined based on the considerations made by the participants of various courses [15]. On the other hand, cooperative/collaborative learning should be a line of development addressed in MOOC designs [16], focused on more interactive pedagogies [17]. Based on the above, these parameters are included in the three quality levels of the UNE standard that must be provided by the educational organizations that offer them. In this way, the three dimensions of satisfaction of this standard are: quality of the recognition factor of training for employability, quality of the learning methodology factor and quality of the accessibility factor. Information on the quality levels is expressed according to a cumulative star rubric representation system, where one asterisk shows the minimum level and five asterisks represent the maximum level. Therefore, the assessment obtained in each dimension is represented by an equal number (from one to five) of accumulated asterisks starting from the left. Therefore, the quality levels of this standard are cumulative, in such a way that each level is also the sum of the contents of the previous levels.

### 2.3. The EduTool^®^ Assessment Tool

EduTool^®^ is an instrument for assessing the quality offered for virtual courses, capable of analyzing and identifying features of didactic quality in the teaching of virtual courses from three dimensions [18]. This instrument, with a registered trademark at the Spanish Patent and Trademark Office (current file number: 3,087,298), is the result of a doctoral thesis by Dr. D. Miguel-Baldomero Ramírez-Fernández entitled “*Model of rules for the analysis and evaluation of MOOCs with the* [13] *standard for the quality of virtual training*”, which received the extraordinary doctorate award 2015/2016, awarded by the Pablo de Olavide University of Seville and developed at the Computational Intelligence Laboratory (LIC). This instrument was developed with the normative adaptation mentioned above [13], and the LIC analyzed the weights of the factors of the dimensions of the standard on Quality Management of virtual training through fuzzy logic. For all of the above, this model provides the same weighted indicators of the previous normative standard [13] of quality of virtual training, which are also valid for supervising all aspects of any non-face-to-face teaching of non-university educational centers [19].

### 2.4. Key Aspects of the Functions and Attributions of the Education Inspectorate in Spain

In accordance with Organic Law 3/2020 [20], in Spain the inspection, supervision and evaluation of the educational system is the responsibility and competence of public authorities. Therefore, it is up to competent public administrations to order, regulate and exercise educational inspection within the respective territorial scope. In this sense, inspection is carried out on all the elements and aspects of the educational system in order to ensure compliance with the law, the guarantee of rights and the observance of duties of those who participate in teaching and learning processes, the improvement of the educational system, and the quality and equity of teaching.

In line with this, functions of the Educational Inspectorate include supervision, evaluation and control from pedagogical and organizational points of view, the operation of educational centers, as well as the projects and programs that they develop; supervising the teaching practice and managerial function and collaborating in their continuous improvement; participating in the evaluation of the educational system and its elements; and ensuring compliance in educational centers with the laws, regulations and other provisions in force that affect the educational system.

The educational inspection is exercised by educational administrations through public officials of the Corps of Education Inspectors. In accordance with the aforementioned functions, the attributions of the Educational Inspectorate include knowing, supervising and observing all the activities that are carried out in the centers, both public and private, to which they are granted free access; receiving from other officials and those responsible for educational centers and services, public and private, the necessary collaboration for the development of their activities, for whose exercise the inspectors are considered a public authority; and submitting reports and making requirements when non-compliances are detected in the application of the regulations and taking minutes, either by own initiative or at the request of the corresponding administrative authority.

As a conclusion of the functions and powers of the Educational Inspectorate in the national territory of Spain, it can be deduced that the evaluators with the highest performance in the supervision of non-face-to-face teaching in non-university educational centers are education inspectors.

### 2.5. Fuzzy Logic in the Educational Context

Fuzzy logic is an alternative to discrete logic in the sense that degrees of categorical belonging are used instead of ascribing to maximum categories of the opposite order. Hence, it has been forcefully defined as a mode of reasoning that applies multiple truth or confidence values to restrictive categories during problem-solving [21,22]. According to this, affective prediction studies have shown that people have a decision-making bias due to random and systematic errors they make when anticipating their own future emotional states [23]. Taking into account this level of divergence between anticipated reactions to decisions and with this normative standard as a theoretical framework, it is suggested that the use of this type of method avoids these problems. Based on this, educational processes are not always discrete, since there may be many other real possibilities that are not taken into account today, or that are falsely applied to discrete categories. These scenarios are situated before a reductionism that does not conform to the truth, and fuzzy logic could help to propose a language of representation that is more faithful to the reality that is intended to be studied [24]. In this sense, and in line with these authors, complex education is chaotic, that is, uncertain, and fuzzy logic is, at the same time, a strategy for dealing with problems of uncertainty. Thus, in evaluations and other flexible applications of concepts, imprecise terms are introduced into propositions that actually impede typical discrete logic reasoning (usually, in general, probably progresses well, needs improvement, etc.), so the educational theory itself falls into contradiction with its own theoretical approaches.

In almost all educational processes there are, in parallel, multiple blurred values that the theory of education, linear and ordered, does not contemplate. Based on this problem, fuzzy logic affects precisely these issues as it is able to address reasoning in undefined issues [24].

The relationship between discrete or traditional logic and set theory also has its projection in the strong connection between fuzzy logic and fuzzy set theory. Thus, if in classical theory a subset *U* of a set *S* can be defined, as shown in Equation (1), as a relation between the elements of *S* and the elements of the set 0, 1:

(1) U:S→{0,1}

This relation can be represented as a set of ordered pairs, whose first element is an element of the set *S*, and the second an element of the set 0, 1, with exactly one ordered pair for each element of the set *S*. The value zero represents the non-membership of the set, and the value one the complete membership. In this way, sentences of the form “*X* is in *U*” can be evaluated by looking for the ordered pair whose first element is *X*. The truth or falsity of this sentence depends on the value of the second element of the pair (if it is 1 it is true and if it is 0 is false).

Analogously, a fuzzy subset *F* of a set *S* can be defined as a set of ordered pairs, whose first element is an element of the set *S*, and the second element, a value of the interval [0, 1]—closed interval—with exactly one ordered pair for each element of the set *S*. As in the case of the traditional theory, the value 0 indicates the non-membership of the set, and the value 1, the total membership; values between 0 and 1 will establish the degrees of membership of the element in the fuzzy set *F*. Thus, this relation is considered a function, the membership function of the set *F*, so that a sentence of the type “*X* is in *F*” is evaluated by looking among the ordered pairs for the one whose first element is *X*. The degree of truth of this sentence is determined by the value of the second element of the pair.

Therefore, although there does not seem to be much difference with this type of function, it has great applications if one takes into account some examples of fuzzy sets in an educational context, such as, for example, unmotivated students, negligent parents, adolescents in a situation of social precariousness, the quality of the classrooms, the supervision of virtual training, etc. In this sense, it seems difficult to determine a clear border between the membership and non-membership of an element to this type of set [24].

## 3. Methodology

### 3.1. Research Setting

As a result of the COVID-19 action protocol for the 2020/2021 academic year established in Spain, a document on COVID-19 prevention, protection, surveillance and health promotion measures was published, dated 29 June 2020, in which it was determined that a contingency plan for COVID-19 must be developed in educational centers in order to offer a safe school environment to students and staff of educational centers. Along these lines, the instructions of 6 July 2020 from the Vice-Ministry of Education and Sports were published, regarding the organization of educational centers for the 2020/2021 school year, motivated by the COVID-19 health crisis, and in its fifth instruction regulates the elaboration of the protocol. Thus, the educational centers prepared a COVID-19 action protocol so that the teaching–learning processes were carried out safely during the 2020/2021 school year. This protocol considered the following situations:

(a) Situation of face-to-face teaching, adjusted to the specific action protocol.
(b) Exceptional situation with telematic teaching.

In addition, the document “*Protocol of actions in cases of COVID-19 of the Ministry of Health and Families dated 13 August 2020*” alludes to the possibility that both situations occur simultaneously in the same center. The specific action protocol was flexible, subject to the evolution of the health crisis and valid for the 2020/2021 academic year.

For all of the above, the study scenario of the research presented focuses on the line of training carried out in December 2020 by the General Inspectorate of Andalusia (autonomous community of Spain), on “supervision and technical advice -normative of the processes of non-university centers related to digital transformation”, within the framework of the training plan for the improvement and professional updating of the Educational Inspectorate of Andalusia for the academic year 2020/2021, within the lines of action training “Promotion of knowledge, dissemination and exchange of innovative practices in the field of Education Inspection” and “Dissemination among the Education Inspection of experiences and successful innovative practices in the field of education”. All this resulted in the training activities: innovative methodologies and organization models; classroom observation as a tool to measure what occurs in the classroom, both in terms of the time spent learning and the quality of teaching practices; and the platforms and digital resources available to the centers, according to the resolution of 27 July 2020 by the Vice-Ministry, which approves the instructions for the development, direction and coordination of the general action plan of the Education Inspectorate and the training plan for professional improvement and updating for the 2020/2021 school year within the quadrennial framework of the order of 29 July 2019, which establishes the general action plan of the educational inspection Andalusia for the period 2019–2023.

### 3.2. Methodology in the Configuration of the Indicators to Supervise the Quality Aspects of Non-Face-to-Face Teaching in Non-University Educational Centers

In order to supervise non-face-to-face teaching in non-university educational centers, three virtual teaching assessment tools and the key aspects of the functions and attributions of the Educational Inspectorate of Spain mentioned above were selected. Taking into account the considerations of [25], the choice of tools and key aspects of the Educational Inspectorate was decided in an intentional and reasoned way; that is, based on the criteria previously established by the researcher, not at random, selecting indicators that could best respond to the objectives of the research study and help to understand, discover and interpret the phenomenon studied in depth [26]. Likewise, in accordance with [27], it can be indicated that the type of sampling of virtual course evaluation instruments was a varied sample, since the aim was to document diversity to look for differences and coincidences, patterns and particularities. In summary, a criterial and intentional sampling approach was used [28]; that is, the most relevant evaluation instruments of virtual courses available in the research literature and the most important aspects of the functions and attributions of the Inspectorate were selected, namely education in the national territory and evaluation of agents with greater performance in the supervision of non-face-to-face teaching in non-university educational centers.

Content validity consists of how adequate the sampling that makes a test of the universe of possible behaviors is according to what it is intended to measure [29]. According to [30], content validity tries to determine to what extent the items of an instrument are representative of the domain or content universe of the property to be measured, and regarding the procedure, he assures that it is a question of judgement. That is, content validity is generally estimated subjectively or intersubjectively. The most commonly used procedure to determine this type of validity is what is known as expert judgment [31]. In agreement with this, [32] argues that expert judgment in many areas is an important part of the information when experimental observations are limited.

Based on the above, for this evaluation indicator collection procedure for the SULODITOOL^®^ instrument, a trademark registered with the Spanish Patent and Trademark Office (pending application number M4177803), a panel of 4 expert coders was configured: a computer engineer and doctor of educational sciences, a bachelor of pedagogy, a bachelor of classical philology, geography and history, and a bachelor of philosophy and a doctor of humanities; all education inspectors, and assessed the quality indicators for the supervision of non-face-to-face teaching in non-university educational centers of the SULODITOOL^®^ supervision instrument. These have comfortably met the requirements of cultural level, training and knowledge of the theoretical framework of coding work, that is, adequately and jointly assessing the indicators of the three virtual course assessment tools studied and the key aspects of the functions and attributions of the Educational Inspectorate for the configuration of new assessment indicators of the innovative instrument under study. This process was carried out from April to June 2021.

The criteria on the quality of the analysis of the coding of the new indicators are based on its validity and reliability. Thus, the importance of the reliability of the configuration of these indicators stems from the security offered by this procedure in that the indicators have been obtained independently of the encoder that measures them. In other words, the encodings are reliable because they remain constant in all the variations of the indicator configuration process by expert judgement.

In this way, reliability establishes limits to the potential validity of the indicators resulting from the research and does not guarantee their validity. Therefore, the validity of this work has clear criteria for validating the results obtained, so that other researchers can collect the appropriate evidence and check whether the inferences produced are accurate. In this research, the requirement of external and indicator-oriented validity was taken into account, since to what extent the coding of the configuration of the indicators of an instrument or analysis tool is representative of the information inherent in the data was evaluated, along with available data on the supervision of non-face-to-face teaching in non-university educational centers. To justify this validity, indicator by indicator has been sampled until finding a representative sample of 10 quality indicators for the supervision of said teaching. Once this number of indicators has been achieved, it can be said that good sampling validity has been obtained in this selection analysis, since possible additional indicators would provide very similar assessments of quality in said teaching by the coders.

Table 1 shows the configuration of the ten selected indicators of the SULODITOOL^®^ instrument for the analysis of the supervision of non-face-to-face teaching in non-university educational centers.

### 3.3. Assessments of the Weights of the Indicators for the Analysis of the Supervision of Non-Face-to-Face Teaching in Non-University Educational Centers by Expert Judges

The weights of the indicators were analyzed using fuzzy logic. In this sense, and as previously mentioned, content validity consists of how adequate the sampling that tests the universe of possible behaviors is, according to what is intended to be measured [29]. According to [30], content validity attempts to determine to what extent the weights of the instrument items are representative of the domain or content universe of the property to be measured, and regarding the procedure, he ensures that it is a matter of judgment. That is, content validity is generally estimated subjectively or intersubjectively. The most commonly used procedure to determine this type of validity is what is known as expert judgment [31]. In agreement with this, [32] argues that expert judgment in many areas is an important part of the information when experimental observations are limited.

For the assessment of the weights of the indicators of the instrument, 242 expert judges were selected, all of them education inspectors of the different territorial delegations of the Ministry of Education and Sports of the Junta de Andalucía, and with extensive professional experience in the supervision, evaluation and advising of the Spanish educational system. In this regard, the intentional selection of the judges ensured that the participants were experts on a relevant topic [33] in order accurately to assess the importance of each indicator in the quality of non-face-to-face teaching in non-university educational centers. Table 2 shows the scale of importance of the indicators in the range between “extremely low” and “extremely high”.

The research was designed for 6 different scenarios for 3 types of non-university education (early childhood education, compulsory secondary education and post-compulsory education) and 2 virtual teaching modalities (asynchronous and synchronous), as shown in Table 3.

The membership functions are the fuzzy set that expresses the degree of membership to the set that each of the elements has. In this sense, the fuzzy set *A* in *X* can be defined, as shown in Equation (2), as the set of ordered pairs:

(2) $$A=\left\{\left(x,\mu_{A}(x)\right)|x\in X\right\}$$

where *μ_A_*(*x*) is the fuzzy set membership function. This membership function associates for each element of *X* (opinion of the expert on the scale of linguistic variables) a degree of membership to the set *A*. The value of this function is in the interval between 0 and 1, with 1 being the value for maximum membership and 0 for none. In the case of expert data, the function used is of type Π (bell-shaped or Gaussian). As shown in Equation (3), the Gaussian symmetric function depends on two parameters: σ (standard deviation) and c (mean).

(3) $$f(x;\sigma ,c)=e^{\frac{-{\left(x-c\right)}^{2}}{2\sigma^{2}}}$$

Thus, in the set of Equation (4) the linguistic variables of the experts are defined.

(4) Extremely low (EL) = f(x; 0.139, 0) Very low (VL) = f(x; 0.139, 0.111) Low (L) = f(x; 0.139, 0.222) Medium low (ML) = f(x; 0.139, 0.333) Little low (LL) = f(x; 0.139, 0.444) Little high (LH) = f(x; 0.139, 0.555) Medium high (MH) = f(x; 0.139, 0.666) High (H) = f(x; 0.139, 0.777) Very high (VH) = f(x; 0.139, 0.888) Extremely high (EH) = f(x; 0.139, 1)

**Figure 1:**
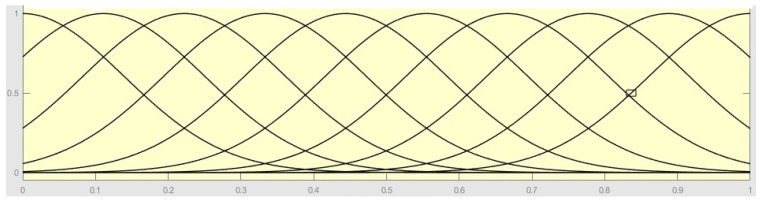
Aggregation function of the membership functions of the linguistic variables of the experts.

The aggregation of the linguistic variables of the experts were treated using the “Probabilistic OR” function. This function returns the probabilistic OR, also known as the “algebraic sum”. In this way, if *x* has two columns as *x* = [a; b], then *y* = a + b − ab; and if *x* has only one column, then *y* = *x*. In the research scenario, Figure 1 represents the aggregation of the membership functions of the linguistic variables of the experts.

The experts’ data are based on fuzzy sets and these data originate from the use of linguistic qualifications. After forming the fuzzy sets corresponding to this study, it is necessary to obtain an answer to these interpretations. In this way, defuzzification consists of going from a fuzzy response to one that is not. Due to the characteristics of this scenario, the area centroid method was used. Thus, a defuzzified value of a membership function associated with the value of the variable x was returned using the defuzzification strategy of Formula (5).

(5) $$Centroide=\frac{\int_{0}^{1}f(x)xdx}{\int_{0}^{1}f(x)dx}$$

As an example, in Equation (6) the defuzzified value of indicator 1 of scenario 6 was particularized, that is, non-face-to-face teaching in post-compulsory education with synchronous modality.


(6) $$\frac{\begin{array}{l} \int_{0}^{1}71\cdot x\cdot e^{\frac{-{\left(x-0\right)}^{2}}{2\cdot 0.139^{2}}}dx+\int_{0}^{1}26\cdot x\cdot e^{\frac{-{\left(x-0.111\right)}^{2}}{2\cdot 0.139^{2}}}dx+\int_{0}^{1}17\cdot x\cdot e^{\frac{-{\left(x-0.222\right)}^{2}}{2\cdot 0.139^{2}}}dx+\int_{0}^{1}14\cdot x\cdot e^{\frac{-{\left(x-0.333\right)}^{2}}{2\cdot 0.139^{2}}}dx+\int_{0}^{1}20\cdot x\cdot e^{\frac{-{\left(x-0.444\right)}^{2}}{2\cdot 0.139^{2}}}dx+\\ \int_{0}^{1}21\cdot x\cdot e^{\frac{-{\left(x-0.555\right)}^{2}}{2\cdot 0.139^{2}}}dx+\int_{0}^{1}18\cdot x\cdot e^{\frac{-{\left(x-0.666\right)}^{2}}{2\cdot 0.139^{2}}}dx+\int_{0}^{1}18\cdot x\cdot e^{\frac{-{\left(x-0.777\right)}^{2}}{2\cdot 0.139^{2}}}dx+\int_{0}^{1}18\cdot x\cdot e^{\frac{-{\left(x-0.888\right)}^{2}}{2\cdot 0.139^{2}}}dx+\int_{0}^{1}19\cdot x\cdot e^{\frac{-{\left(x-1\right)}^{2}}{2\cdot 0.139^{2}}}dx \end{array}}{\begin{array}{l} \int_{0}^{1}71\cdot e^{\frac{-{\left(x-0\right)}^{2}}{2\cdot 0.139^{2}}}dx+\int_{0}^{1}26\cdot e^{\frac{-{\left(x-0.111\right)}^{2}}{2\cdot 0.139^{2}}}dx+\int_{0}^{1}17\cdot e^{\frac{-{\left(x-0.222\right)}^{2}}{2\cdot 0.139^{2}}}dx+\int_{0}^{1}14\cdot e^{\frac{-{\left(x-0.333\right)}^{2}}{2\cdot 0.139^{2}}}dx+\int_{0}^{1}20\cdot e^{\frac{-{\left(x-0.444\right)}^{2}}{2\cdot 0.139^{2}}}dx+\\ \int_{0}^{1}21\cdot e^{\frac{-{\left(x-0.555\right)}^{2}}{2\cdot 0.139^{2}}}dx+\int_{0}^{1}18\cdot e^{\frac{-{\left(x-0.666\right)}^{2}}{2\cdot 0.139^{2}}}dx+\int_{0}^{1}18\cdot e^{\frac{-{\left(x-0.777\right)}^{2}}{2\cdot 0.139^{2}}}dx+\int_{0}^{1}18\cdot e^{\frac{-{\left(x-0.888\right)}^{2}}{2\cdot 0.139^{2}}}dx+\int_{0}^{1}19\cdot e^{\frac{-{\left(x-1\right)}^{2}}{2\cdot 0.139^{2}}}dx \end{array}}=0.467$$

In Equation (7), the normalization equation of the defuzzified values of the obtained indicators is presented.

(7) $$X_{normalized}=\frac{X-X_{\min}}{X_{\max}-X_{\min}}+1$$

Based on the calculations made, Table 4 shows the normalized weightings of the 10 selected indicators in the 6 scenarios proposed by the 242 expert judges and by means of fuzzy logic.

The universe was segregated into diffuse regions according to the degree of weighting of each indicator. This process was designed by a panel of the 4 previous expert coders, all education inspectors who comfortably met the requirements of cultural level, training and knowledge of the theoretical framework for the work of segregation of the universe and for the configuration of the classification of instrument indicators. In this way, the indicators were classified as HW (high weight), MW (medium weight) and LW (low weight), depending on the weighting band in which it was found. In this sense, those indicators that had a weight greater than or equal to 11% were be considered as HW, those with a weight between 10% and less than 11% were classified as MW, and those with a percentage less than 10% were LW. Thus, for application scenario 6, Table 5 is used.

The global qualitative assessments of non-face-to-face teaching in non-university centers are: initial (I), basic (Bas), good (G), very good (VG) and excellent (E). In this way, and according to the aforementioned panel of 4 expert coders, each indicator was valued as acquired if at least 75% of its performance had been achieved. The generation of fuzzy rules and the rule base was applied to the global qualitative assessment of the evaluated teaching. In this sense, each of the qualitative assessments of each level of weight indicators (HW, MW and LW) were taken into account. Thus, Table 6 shows some acronyms necessary in the application of fuzzy rules.

**Figure 2:**
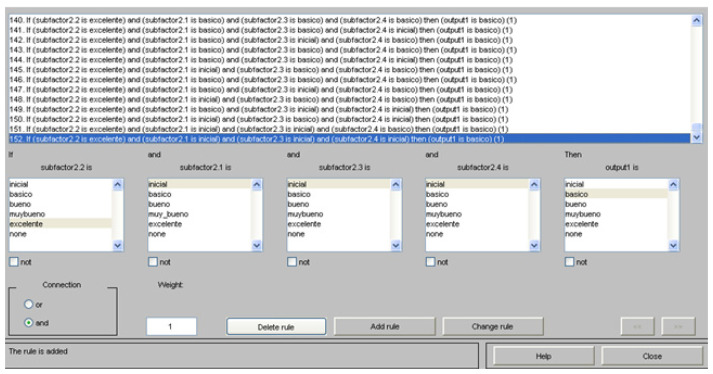
Fuzzy rules system extract. Source: own elaboration through the MatLab^®^ Fuzzy Tool application.

Based on the above, Figure 2 shows an extract of the fuzzy rules system contemplated for the qualitative assessment of the different teachings through the MatLab^®^ Fuzzy Tool application.

## 4. Results

The result of the study defined the SULODITOOL^®^ tool with the following dimensional structure:

1. The 10 dimensions of the evaluation indicators in the supervision of non-face-to-face teaching in non-university educational centers. These dimensions have been designed for indicators of high weight (HW), medium weight (MW) and low weight (LW), according to non-university education and asynchronous or synchronous modality.
2. A quantitative and qualitative assessment model of the quality of distance learning.
3. A report with the deficiencies and proposals for improvement of each dimension by indicators.

Each indicator is dichotomous (yes/no) and measures if at least 75% of its performance is achieved.

The design of instruments in the social sciences must guarantee content validity and reliability. Content validity is the effectiveness with which the tool measures what it is intended to quantify [34,35]. That is, the degree of mastery of the specific content of what is being measured and, therefore, that the selected items are truly indicative of what is to be measured [27].

This research bases the validity of the content of the tool on the bibliographic review carried out and the normative theoretical framework on which it is based (the [13] standard and the supervision regulations for non-face-to-face teaching in Spain). In this sense, it is taken as a premise that this standard complies with the attributes of expert judgment; that is, it is considered an opinion consistent with experience, which is recognized by other researchers in the field, and which provides judgments and reliable assessments [36].

In relation to the reliability of the tool, a measurement is reliable when its results are the same or similar to the same individual or group, regardless of the people who apply the instrument [37]. That is, the reliability of an instrument guarantees the stability, repeatability or accuracy of results [27,28]. In conclusion, this research demonstrates the reliability of the instrument by obtaining the same results when applied by different researchers.

Table 7 presents the SULODITOOL^®^ tool in scenario 6: non-face-to-face primary education with asynchronous modality.

For all these reasons, this complete adaptation of other evaluation instruments and normative standards of quality of virtual training has originated the SULODITOOL^®^ tool for supervision and qualitative and quantitative assessment of non-face-to-face teaching in non-university educational centers. In this line, Table 8 analyzes the weights of the indicators of the six scenarios in their three classifications: HW (high weight), MW (medium weight) and LW (low weight).

Based on the above information, it is evident that indicator 4, that is, the training design and methodology used, should be considered as a key factor in all teaching and modalities, whether synchronous or asynchronous. However, it is evident for all scenarios that the less relevant indicators in supervision are 1, 7, 8 and 10, that is, regulations and procedures, tutoring, collaborative learning and formative evaluation

From another point of view, indicator 9, that is, the configuration of activities, tasks and feedback is considered, with an average weight, as a key factor in the supervision in the synchronous modality of all the teachings. In this line, the same does not occur in asynchronous modality, where it would take a high weight only in post-compulsory education, considering it as medium weight in the rest of the education system.

**Figure 3:**
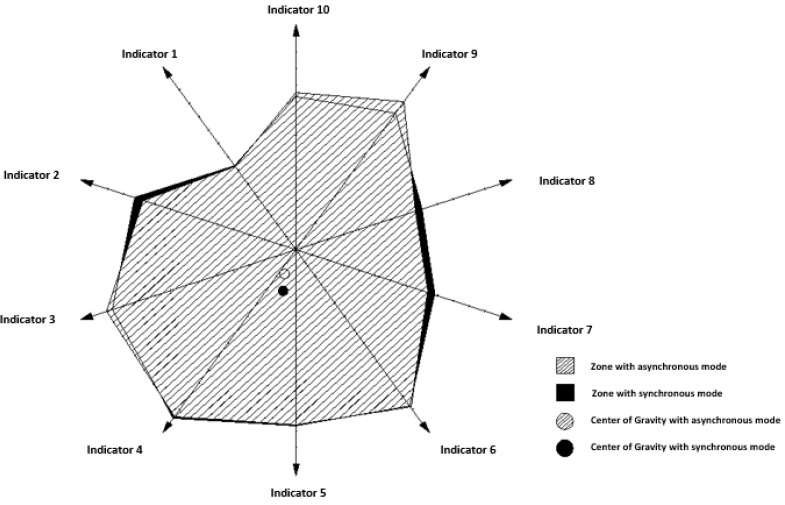
Graphical representation of the area of importance and the center of gravity in the supervision of the indicators of the SULODITOOL^®^ tool in post-compulsory education (scenario 5 and 6). Source: own elaboration through the AutoCAD^®^ application.

If the assessment is focused on a graphic representation of areas of importance in the supervision of the indicators of the SULODITOOL^®^ tool created by the weights of said indicators, as shown in Figure 3, for post-compulsory education in its 2 scenarios, modality synchronous and asynchronous, it was found that the area configured by the weights of the synchronous modality was higher by 0.37% than the area of the asynchronous modality in the teaching of primary education, by 1.09% in the teaching of compulsory secondary education and by 0.20% in post-compulsory education. That is, there is a greater tendency to the importance of the weights of the indicators of the synchronous mode than the asynchronous mode, whose area of importance in supervision becomes smaller.

It is verified in Figure 3 that the center of gravity of the synchronous mode is lower and to the left than that of the asynchronous mode. This situation shows that the weights of indicators 3, 4, 5 and 6 are more relevant in the synchronous modality than the rest of the indicators, and vice versa, than the weights of indicators 2, 1, 10, 9, 8 and 7. They are more influential in the asynchronous modality in the supervision of non-virtual teaching. That is to say, the general quality of the content, the training design and methodology, the motivation and participation, and the learning materials, have a higher degree of importance to the synchronous modality than the rest of the indicators. On the other hand, regulations and procedures, teacher training, tutoring, collaborative learning, activities, tasks and feedback, and formative evaluation have a higher degree of importance in asynchronous modality than the rest of the indicators.

**Figure 4:**
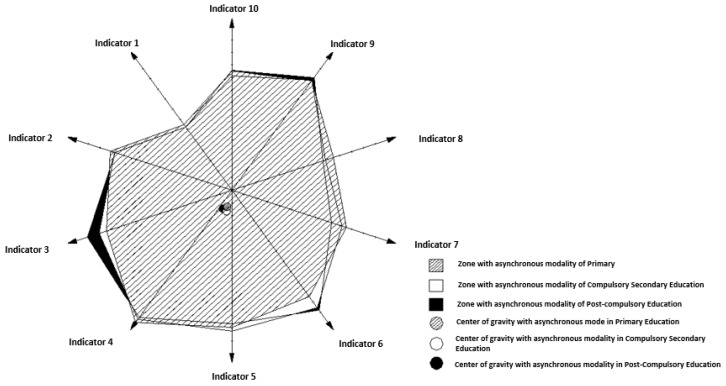
Graphical representation of the area of importance and the center of gravity in the supervision of the indicators of the SULODITOOL^®^ tool in asynchronous mode and in all teaching scenarios (scenarios 1, 3 and 5). Source: own elaboration through the AutoCAD^®^ application.

**Figure 5:**
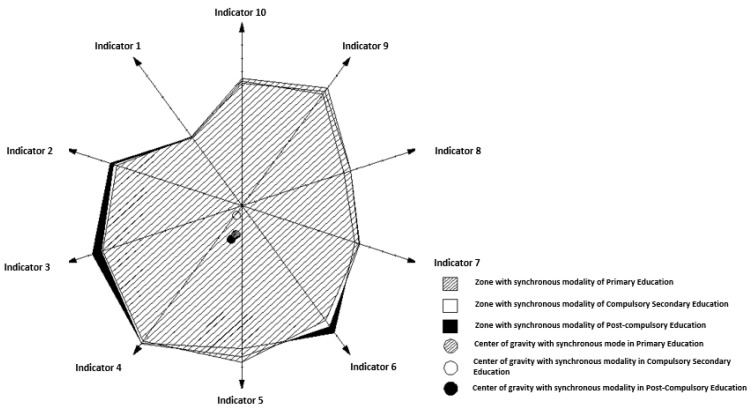
Graphical representation of the area of importance and the center of gravity in the supervision of the indicators of the SULODITOOL^®^ tool in the synchronous mode and in all the teachings (scenarios 2, 4 and 6). Source: own elaboration through the AutoCAD^®^ application.

If the previous graphical analysis is conducted in the asynchronous (Figure 4) and synchronous mode (Figure 5) for all the teachings, it can be seen that in the asynchronous mode all the centers of gravity are very close. That is, the importance of the weights of all the indicators in all the teachings is practically the same, regardless of the teaching.

However, in the synchronous modality (Figure 5), the center of gravity of compulsory secondary education is above the rest of education, and very close to the origin of coordinates. The center of primary education is lower and slightly displaced to the left, and that of post-compulsory education a little more displaced downwards and to the left, with respect to the previous one.

With this situation it is verified that in the supervision of compulsory secondary education with synchronous modality it becomes latent that all the weights of the indicators have the same importance practically. However, the same does not occur in primary education and post-compulsory education, where it is evident that the importance of the weights of indicators 3, 4, 5 and 6 is greater than the rest, and this situation is even more emphasized in post-compulsory education. That is, in these last two teachings, the general quality of the content, the training design and methodology, the motivation and participation, and the learning materials have a higher degree of importance in the synchronous modality than the rest of the indicators.

## 5. Conclusions

In the COVID-19 scenario, it is necessary to use tools to monitor the quality of non-university training. This research designed the SULODITOOL^®^ tool, which is a complete adaptation of other evaluation instruments and regulatory quality standards for virtual training. Said instrument configures the weighting of ten evaluation indicators through fuzzy logic methodology tested in six different scenarios.

As a conclusion of the results obtained, it can be shown that the evaluations obtained help to improve the supervision of remote teaching. In line with this, it has been verified that the “training design” and the “methodology used in training” must be seen as key factors in all teachings and modalities. However, “rules and procedures”, “tutoring”, “collaborative learning” and “formative assessment” are considered for all scenarios as less relevant indicators.

It was also verified that the configuration of “activities, tasks and feedback” is considered, with medium weight, as a key factor in supervision in the synchronous modality of all teaching scenarios. In asynchronous modality, this indicator only acquires a high weighting in post-compulsory education, being considered as medium-weight in the rest of education.

If supervision of the teaching scenarios is configured as a graphic representation of areas of importance designed by the weights of the indicators of the SULODITOOL^®^ tool, it is concluded that the area created by the weights of the synchronous mode is greater than the area of the asynchronous modality in all teachings. That is, the supervision of the synchronous mode must be more intense than the supervision of the asynchronous mode, regardless of the teaching.

On the other hand, in the asynchronous modality, all the centers of gravity of all the areas of importance of the teaching scenarios are very close. Therefore, the importance of the weights of all the indicators in all the teaching scenarios is practically the same in the synchronous modality, regardless of the teaching type.

However, in the synchronous modality, the center of gravity of the area of importance of compulsory secondary education is above the rest of education, and very close to the origin of coordinates. The center of gravity of primary education is lower and slightly displaced to the left, and that of post-compulsory education is slightly more displaced downwards and to the left, with respect to the previous one. With this situation, it is verified that in the supervision of compulsory secondary education with synchronous modality, it becomes latent that all the weights of the indicators have the same importance practically. However, the same does not occur in primary education and post-compulsory education, where it is evident that the importance of the weights of indicators 3, 4, 5 and 6 is greater than the rest, and this situation is even more emphasized in post-compulsory education. That is, in these last two teaching scenarios, the “general quality of the content”, the “training design and methodology”, “motivation and participation” and “learning materials”, have a higher degree of importance than the rest of the indicators and are key in the synchronous modality, as evidenced in other types of studies [38].

For all of the above, in this study, the same conclusions were reached as in other investigations, where it is evidenced that non-face-to-face teaching has a solid pedagogical basis in its various formats [39]. However, although this form of training has made a significant appearance, it is evident that there is a lack of quantitative quality in terms of virtual training. For this, new research paths that open interdisciplinary points of attention and reflection on their deficiencies in those indicators and dimensions analyzed in this research are necessary.

## 6. Discussion

The discussion is the interpretation of the results obtained in light of the research question or the hypothesis, which in this case was about the design of a graphic model on the supervision of virtual teaching in non-university education.

Based on the data obtained, it can be interpreted that the graphic model offers quick information on the importance of the aspects to be supervised in non-contact teaching. In this sense, depending on the situation of the center of gravity with respect to the axes of the indicators, very relevant information is offered on the importance of the indicator in the supervision of the quality of teaching and modality analyzed.

In this way, and to obtain quality virtual teaching, a center of gravity must be obtained that coincides with the origin of the coordinates of the graph. That is to say, in this last situation it would be evident that all the indicators would have the same importance and virtual teaching and face-to-face teaching could be considered practically the same.

In this research, the authenticity of the results obtained is a reflection of the intuition of the Education Inspectorate of Spain. In this work, the conceptual, methodological and empirical phases of the results were examined, and the sample of inspectors was identified as a possible positive factor that could have influenced the results obtained. As a consequence of all this, this confirms the high internal validity and reliability of the study. In the same way, the non-selection of higher university virtual education is also evidenced as a possible limitation of the study. Based on the results obtained, it is recommended that virtual university teaching scenarios be analyzed as suggestions for future research and that the sample be expanded with a greater number of judges who are experts in remote teaching supervision.

On the other hand, in the bibliographical review, no conclusions or results obtained to compare this work with other investigations that have studied the same phenomenon were found. In line with this, it would have been possible to identify similar findings and those that are different in the field of supervision of virtual non-university education.

The contribution of the research to the future lines of supervision of the quality of non-face-to-face teaching in future pandemic scenarios or any other scenario that requires virtual teaching and that adjusts to the needs of the users is also highlighted. In this way, the disadvantages of physical absence would be limited, and it would come closer and closer to the advantages of face-to-face teaching.

Finally, although there are research works that include a comparative analysis of the main virtual platforms and the construction and validation of an instrument to measure the perception of quality of non-contact teaching [40,41], in any case, the evaluation of the quality of virtual training is on the research agenda for the future with a view to efficient supervision.

A greater number of studies on some quality indicators of virtual teaching is necessary, in addition to longitudinal [42] or comparative [43] studies. In addition, research should continue to answer questions about methods that improve the validity and reliability of the evaluations of the supervisors or about techniques that guarantee systems of objective evaluations in a simple way, and how they can be integrated into open learning environments [44] to provide more guarantee of usability and solvency to the designs of future quality instruments.

## Figures and Tables

**Table 1:** Selected indicators of the SULODITOOL^®^ instrument for the analysis of the supervision of virtual teaching in non-university educational centers.

Indicator Number	Indicator Name
1	Regulations and procedures
2	Teacher training
3	Overall content quality
4	Training design and methodology
5	Motivation and participation
6	Learning materials
7	Tutoring
8	Collaborative learning
9	Activities, tasks and feedback
10	Formative evaluation

**Table 2:** Scale of importance of the experts when completing the form.

Quantitative Scale of Importance of the Indicators	Qualitative Scale of Importance of the Indicators
1	“extremely low”, hereafter EL
2	“very low”, hereinafter VL
3	“low”, hereafter L
4	“medium low”, hereafter ML
5	“little low”, hereafter LL
6	“little high”, hereafter LH
7	“medium high”, hereafter MH
8	“high”, hereafter H
9	“very high”, hereinafter VH
10	“extremely high”, hereafter EH

**Table 3:** Scenarios designed for non-university education for the collection of information from the expert judges through the completion of the form.

Scenarios	Non-University Education and Virtual Teaching Modalities
1	Non-face-to-face teaching in primary education with asynchronous modality
2	Non-face-to-face teaching in primary education with synchronous modality
3	Non-face-to-face teaching in compulsory secondary education with asynchronous modality
4	Non-face-to-face teaching in compulsory secondary education with synchronous modality
5	Non-face-to-face teaching in post-compulsory education with asynchronous modality
6	Non-face-to-face teaching in post-compulsory education with synchronous modality

**Table 4:** Values of the weights of the instrument indicators in each scenario.

Indicators	Scenario 1	Scenario 2	Scenario 3	Scenario 4	Scenario 5	Scenario 6
1. Regulations and procedures	6.52	6.34	6.39	6.36	6.29	6.35
2. Teacher training	10.12	9.79	9.86	10.05	9.80	10.32
3. Overall content quality	10.45	10.81	11.09	11.01	12.09	11.72
4. Training design and methodology	13.05	12.69	12.77	12.72	12.59	12.69
5. Motivation and participation	10.92	11.70	11.22	11.23	10.64	10.66
6. Learning materials	10.54	10.68	11.59	11.23	11.84	11.78
7. Tutoring	9.60	9.13	9.20	9.14	8.37	8.86
8. Collaborative learning	8.50	8.46	7.86	8.45	7.63	8.00
9. Activities, tasks and feedback	10.78	10.87	10.85	10.52	11.07	10.29
10. Formative evaluation	9.52	9.53	9.17	9.28	9.67	9.32
Addition	100	100	100	100	100	100

**Table 5:** Classification of the indicators of the instrument according to their in post-compulsory education and with synchronous modality (scenario 6).

Classification of Indicators	Indicators	Percentage or Weight of the Indicator
HW ≥ 11%	3. Overall content quality	11.72%
	4. Training design and methodology	12.69%
	6. Learning materials	11.78%
MW < 11% y ≥ 10%	2. Teacher training	10.32%
	5. Motivation and participation	10.66%
	9. Activities, tasks and feedback	10.29%
LW < 10%	1. Regulations and procedures	6.35%
	7. Tutoring	8.86%
	8. Collaborative learning	8.00%
	10. Formative evaluation	9.32%

**Table 6:** Acronyms used in the application of fuzzy rules.

Acronym	Meaning
N	Level of qualitative assessment of teaching
E	Excellent Level
VG	Very good level
G	Good level
Bas	Basic level
I	Initial level

**Table 7:** SULODITOOL^®^ instrument adapted to scenario 6 of non-face-to-face post-compulsory education with synchronous modality.

Assessment of Non-Face-to-Face Teaching in Post-Compulsory Education and with a Synchronous Modality (Scenario 6)					
Qualitative Assessment of Teaching	Acquisition		Satisfaction Indicators	Assessment	Weight
	Scope	Level			
□Excellent □Very good	□	At least 75%	3. Overall content quality	Yes □ No □	11.72%
	□	At least 75%	4. Training design and methodology	Yes □ No □	12.69%
	□	At least 75%	6. Learning materials	Yes □ No □	11.78%
□Good	□	At least 75%	2. Teacher training	Yes □ No □	10.32%
	□	At least 75%	5. Motivation and participation	Yes □ No □	10.66%
	□	At least 75%	9. Activities, tasks and feedback	Yes □ No □	10.29%
□Basic □Initial	□	At least 75%	1. Regulations and procedures	Yes □ No □	6.35%
	□	At least 75%	7. Tutoring	Yes □ No □	8.86%
	□	At least 75%	8. Collaborative learning	Yes □ No □	8.00%
	□	At least 75%	10. Formative evaluation	Yes □ No □	9.32%
Summary of Teaching Evaluations					
Qualitative assessment of teaching:	□		Initial	Quantitative assessment of teaching:	
	□		Basic		
	□		Good		
	□		Very good		
	□		Excellent		

**Table 8:** Distribution of indicators according to weights in the study scenarios.

	Primary Education		Compulsory Secondary Education		Post-Compulsory Education	
	Asynchronous (Scenario 1)	Synchronous (Scenario 2)	Asynchronous (Scenario 3)	Synchronous (Scenario 4)	Asynchronous (Scenario 5)	Synchronous (Scenario 6)
HW	4	4 5	3 4 5 6	3 4 5 6	3 4 6 9	3 4 6
MW	2 3 5 6 9	3 6 9	9	2 9	5	2 5 9
LW	1 7 8 10	1 2 7 8 10	1 2 7 8 10	1 7 8 10	1 2 7 8 10	1 7 8 10
